# Physiology-Based Pharmacokinetic Modeling of Ropivacaine After External Oblique Intercostal Plane Block in Open Liver Surgery Patients

**DOI:** 10.3390/ph19030348

**Published:** 2026-02-24

**Authors:** Jiali Tang, Jiarui Chen, Ning Sheng, Bowen Zheng, Li Xu, Jinlan Zhang

**Affiliations:** 1 Department of Anesthesiology, Peking Union Medical College Hospital, Chinese Academy of Medical Sciences & Peking Union Medical College, Beijing 100730, China; tangjiali@pumch.cn; 2 State Key Laboratory of Bioactive Substance and Function of Natural Medicines, Institute of Materia Medica, Chinese Academy of Medical Sciences & Peking Union Medical College, Beijing 100050, China; chenjiarui@imm.ac.cn (J.C.); shengcc@imm.ac.cn (N.S.); zhengbowen@imm.ac.cn (B.Z.)

**Keywords:** external oblique intercostal plane block, ropivacaine, metabolites, open liver surgery, PBPK, pharmacokinetics

## Abstract

**Background/Objectives**: The external oblique intercostal (EOI) plane block shows promise for postoperative analgesia after open liver surgery. Pharmacokinetic profiles of ropivacaine after EOI plane block remain unclear. Meanwhile the pharmacokinetic data informs safety assessment, guides post-block monitoring duration, and predicts blockade duration. This study aimed to characterize ropivacaine pharmacokinetics and propose a safe dosing regimen. **Methods**: In this prospective study, patients undergoing open liver surgery received a unilateral single-shot ultrasound-guided EOI plane block with 30 mL of 0.375% ropivacaine. Plasma ropivacaine concentrations were measured to define pharmacokinetics, identify influencing factors, and develop physiology-based pharmacokinetic (PBPK) models for dose optimization. **Results**: Twenty-eight patients (Child-Pugh A, ≤3 liver segments resected) were included. Peak plasma ropivacaine concentration occurred at 10 min and remained below the toxic threshold in all patients. No adverse events were observed. Demographic and surgical factors did not significantly affect pharmacokinetics. The PBPK model-predicted safe doses of ropivacaine were comparable across age groups and relatively high. **Conclusions**: A single-shot EOI plane block with ropivacaine is safe for patients undergoing open liver surgery (Child-Pugh A) with limited resection (≤3 segments). This study provides critical pharmacokinetic data and validated PBPK model, guiding safe dosing to reduce toxicity risks.

## 1. Introduction

Open liver surgery is associated with substantial postoperative pain, with 66% of patients experiencing moderate-to-severe acute postoperative pain [1]. Effective pain management facilitates early postoperative mobilization, reduces stress response and complications, decreases narcotic consumption, and shortens hospital stays [2]. The external oblique intercostal (EOI) plane block is a relatively novel and more effective fascial plane block. It targets the anterior and lateral upper abdominal wall (T6-T10) [3], matching the sensory area of incisions in typical open liver surgery [4].

Ropivacaine is widely used in various fascial plane block techniques. It is high protein-bound (approximately 94% to α1-acid glycoprotein (AAG) in humans) and extensively metabolized in the liver [5]. Cytochrome P-450 (CYP) 1A2 converts it to 3-hydroxy-ropivacaine (3-OH ropivacaine), while CYP3A4 mainly produces 2′,6′-pipecoloxylidide (PPX) [6]. The neurotoxicity of PPX is 1/12 that of ropivacaine [6]. Ropivacaine pharmacokinetics vary markedly in different fascial plane block techniques [7]. However, its pharmacokinetic alterations after EOI plane block remain unclear. This knowledge gap is clinically critical, as the high-dose ropivacaine fascial block in these patients increases the risk of local anesthetic systemic toxicity (LAST) [8,9,10,11,12]. This elevated risk arises from large administered does and impaired hepatic function caused by intermittent hepatic inflow occlusion and liver resection. Therefore, the pharmacokinetic study of ropivacaine and its main metabolites (3-OH ropivacaine and PPX) is essential for precision dosing and toxicity prevention.

From 1968 to 2010, reports of ropivacaine-associated deaths showed a decreasing trend. Since 2010, however, the reporting odds ratio for ropivacaine-related deaths has remained stable, with no further reduction in ropivacaine-induced LAST cases. Measures should be taken to reduce ropivacaine-associated LAST [13]. Physiologically based pharmacokinetic (PBPK) modeling is a promising approach for predicting drug pharmacokinetics and supporting optimal and safe medicine, particularly in special populations [14,15]. PBPK models have been successfully applied to predict drug exposure in patients undergoing liver resection or those with cirrhosis [16,17].

This study aimed to characterize the pharmacokinetics of ropivacaine and its main metabolites (3-OH ropivacaine and PPX) in patients undergoing open liver surgery after EOI plane block, explore factors affecting the pharmacokinetics, and propose a novel ropivacaine dosing regimen based on developed PBPK models. These findings will provide clinical support for the safe application of EOI plane block.

## 2. Results

### 2.1. Pharmacokinetics of Ropivacaine and Its Metabolites

Blood samples were collected from 35 patients, and all were analyzed for ropivacaine concentration. Seven patients were excluded from the pharmacokinetics analysis due to incomplete sample sets that prevented generation of full pharmacokinetic profiles. Finally, samples from 28 patients were included in the pharmacokinetic analyses (Figure S1, Supplementary File S2). The mean patient age was 59.68 [SD 13.21] years. All patients were classified as Child-Pugh grade A, with no cases of renal insufficiency. Detailed demographic characteristics were shown in Table 1.

Individual differences in plasma ropivacaine concentrations were significant. The median peak concentration (C_max_) was 792.56 ng/mL, and the median time to C_max_ (T_max_) was 10 min after ropivacaine administration. The maximum individual concentration reached 2343.78 ng/mL, occurring at 15 min after ropivacaine administration. Pharmacokinetic parameters of ropivacaine are shown in Table 2.

**Figure 1:**
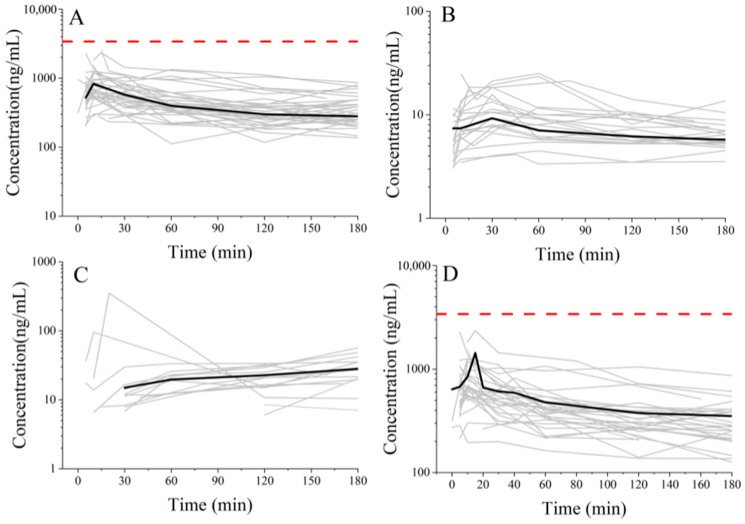
Plasma concentration curves of ropivacaine and its metabolites in each patient. (**A**) shows ropivacaine plasma concentration (*n* = 28). (**B**) shows 3-OH ropivacaine plasma concentration (*n* = 24). (**C**) shows PPX plasma concentration (*n* = 14). (**D**) shows plasma concentration curves of ropivacaine plus metabolites. The red dash line indicates the ropivacaine toxic threshold. Black line indicates the mean concentration.

The peak concentration of ropivacaine plus metabolites, calculated according to toxicity, did not exceed the toxic threshold of ropivacaine (3.4 mg/L) in arterial plasma [18], as shown in Figure 1D. Moreover, no LAST cases occurred among all 35 patients after EOI plane block.

After excluding samples with concentrations below the lower limit of quantification (LLOQ), 25 patients were included in the final analysis for 3-OH ropivacaine and 14 for PPX. Total plasma concentrations of two metabolites were low, as shown in Figure 1 and Table 2. The maximum individual concentration of 3-OH ropivacaine and PPX were 50.81 ng/mL (at 10 min after injection) and 351.50 ng/mL (at 20 min after injection), respectively, as shown in Table 2.

### 2.2. Factors Affecting Ropivacaine Pharmacokinetics

Spearman’s correlation analysis indicated that weight, height, body mass index (BMI), age, sex, hemoglobin, and preoperative albumin were not correlated with pharmacokinetic parameters of ropivacaine (Table 3). Multiple linear regression analysis results showed that the number of hepatic resection segments and the hepatic inflow occlusion duration were not associated with pharmacokinetic parameters of ropivacaine (Table 3).

### 2.3. Development and Verification of Ropivacaine PBPK Models

**Figure 2:**
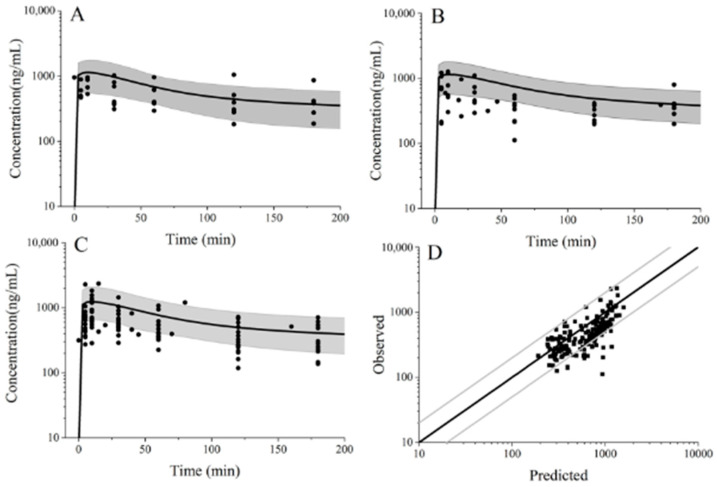
Ropivacaine population pharmacokinetic validated profile of PBPK model in patients undergoing open liver surgery after EOI block. The dosage of ropivacaine was 112.5 mg. The black circle was clinical observed data. (**A**–**C**) represents age from 18 to 45, age from 46 to 59, age over 60, respectively. (**D**) is goodness-of-fit ropivacaine plasma concentration of patients undergoing open liver surgery after EOI block. The grey line shows the 2-fold error range. The black dash shows what was actually tested data from samples.

Individual ropivacaine PBPK models were developed for patients undergoing open liver surgery. Figure 2D illustrates the goodness-of-fit of the model by comparing simulated and clinically observed plasma ropivacaine concentrations. The average fold error (AFE) and absolute average fold error (AAFE) for ropivacaine were 1.34 and 1.55, respectively. Consistent with regulatory guidelines, the model was deemed pharmacokinetically acceptable, as both AFE and AAFE fell within the 0.5–2.0 range [19].

Stratified validation by age cohort further demonstrated the robustness of the models across subgroups (18–45 years: AFE= 1.60, AAFE= 1.76; 45–59 years: AFE= 1.46, AAFE= 1.54; 60–79 years: AFE= 1.27, AAFE= 1.49). This validation verified the model’s ability to capture age-related physiological variations.

To further assess model reliability, simulated ropivacaine pharmacokinetic values were compared with clinical observations. As shown in Table S1 (Supplementary File S2), all simulated pharmacokinetics results fell within a 2-fold error range in different age groups. Therefore, the PBPK model was considered suitable for describing the pharmacokinetics of ropivacaine in patients undergoing liver surgery after EOI plane block and can serve as a foundation for population PBPK modeling.

The simulated population ropivacaine plasma concentration-time profiles were in conformity with the observed data, as shown in Figure 2.

### 2.4. PBPK Sensitivity Analysis

The sensitivity parameter analysis showed that C_max_ increased with elevated EOI plane blood flow and distribution coefficient of fascia and blood (K_f,b_), but decreased with higher fraction unbound (f_up_) and greater adipose volume at the EOI plane (Figure S2 in Supplementary File S2). Increases in EOI plane blood flow, the Michaelis–Menten constant (K_m_), and K_f,b_, and decreases in f_up_, liver volume, and maximum reaction velocity (V_max_) increased the area under the curve (AUC). Increases in K_m_ and decreases in f_up_, liver volume, and V_max_ reduced clearance (CL). Notably, CYP enzyme activity parameters exerted a greater impact than liver volume (Figure S2 in Supplementary File S2). It is indicated that ropivacaine exposure and clearance may be more affected by altered enzyme activity. Increases in EOI plane adipose compartment volume and f_up_, and decreases in EOI plane blood flow, increased T_max_.

### 2.5. Simulation Dose Exploration for EOI Plane Block

**Figure 3:**
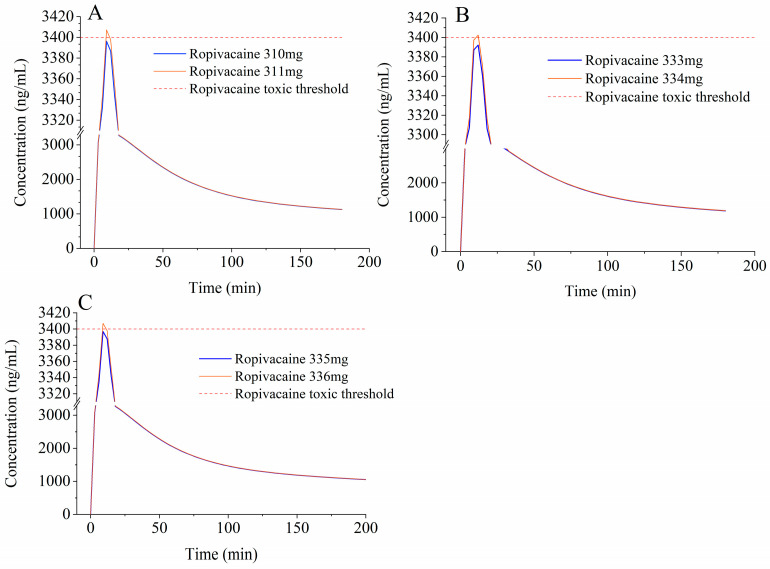
Population pharmacokinetic profile of model-predicted ropivacaine in patients undergoing open liver surgery after EOI block in different dosages. (**A**–**C**) represent age from 18 to 45, age from 46 to 59, age from 60 to 79, respectively. The red dotted line shows the ropivacaine toxic threshold (3.4 mg/L).

Sensitivity parameter analysis showed that a larger EOI plane adipose volume increased C_max_. Because EOI plane adipose volume differs across age groups, ropivacaine dosing regimens for patients undergoing open liver surgery were prospectively established for different age groups based on the developed models. The dosing regimens were determined using simulated ropivacaine C_max_, as shown in Figure 3. Knudsen et al. reported that the minimum total arterial concentration of ropivacaine in patients with toxic symptoms was 3.4 mg/L [18]. In addition, many studies regard 3.4 mg/L as the total ropivacaine toxic arterial plasma threshold [20].

To avoid the risk of LAST (3.4 mg/L as ropivacaine toxic threshold), our simulations suggested that total ropivacaine doses might be considered not to exceed 335 mg for patients aged 18–45 years, 333 mg for those aged 46–59 years, and 310 mg for patients aged 60–79 years (Table 4). It should be emphasized that these dose limits are derived from model-based explorations and are intended primarily to inform future clinical study design.

## 3. Discussion

To our knowledge, this is the first investigation to characterize the pharmacokinetics of ropivacaine and its metabolites after EOI plane block in patients undergoing liver resection and provide dosage recommendations based on PBPK models (Table 5).

Our study indicated that median T_max_ of ropivacaine was 10 min after EOI plane block. This T_max_ was shorter than rectus abdominis sheath block, transversus abdominis plane block (TAPB) (18–60 min) [7], and quadratus lumborum block (35 min) [21]. However, it was similar to that for paravertebral block (7.5 min) [22], erector spinae plane block (10 min) [11], and intercostal nerve block (10 min) [23]. The CL of ropivacaine under EOI plane block is higher than TAPB (0.79 L/min vs 0.15 L/min) [24] and paravertebral block (0.79 L/min vs 0.48 L/min) and is similar to intercostal nerve block (0.79 L/min vs 0.65 L/min). Therefore, following EOI plane block, the pharmacokinetics of ropivacaine exhibited a rapid absorption and fast clearance, suggesting that routine prolonged monitoring after block may not be mandatory in these patients.

The active metabolites of ropivacaine include 3-OH ropivacaine and PPX. The fraction of ropivacaine metabolized to PPX ranges from 5% to 15% [25,26], and it may increase during postoperative infusion [27]. PPX is prone to accumulate due to its lower clearance and longer elimination half-life relative to ropivacaine. Although the neurotoxicity of PPX is one-twelfth that of ropivacaine [6], after prolonged infusion via a blocking catheter, PPX could significantly contribute to systemic toxicity [27]. Meanwhile, in patients with impaired liver function [6,26], the combined exposure of large volume local anesthetic and active metabolite accumulation may exceed the toxicity threshold easily. Thus, we measured concentrations of 3-OH ropivacaine and PPX. Our results indicated the proportion of ropivacaine metabolites in plasma remained relatively low. The fractions of ropivacaine metabolized to 3-OH ropivacaine and PPX were 2.71% and 7.42%, respectively. These values are consistent with reports in patients without hepatectomy (PPX: 5–15%, 3-OH ropivacaine: 2.6–24.5%) [25,26,28]. Our results also indicated that metabolite levels were not significantly affected in patients with resection of less than three liver segments. The low fractions and short half-lives of metabolites suggested a low risk of toxicity due to active metabolite accumulation after a single injection of EOI plane block, while ropivacaine remains the primary contributor to systemic toxicity in this setting. Metabolite monitoring after a single-shot EOI plane block seems unnecessary. In our study, the number of hepatic resections and duration of hepatic blood flow occlusion were not associated with CL; this is possibly due to the limited extent of resection (≤3 segments) and short occlusion time (median 14 min). This supported relative safety of ropivacaine use in such patients. Research stated loss 50% proportion of liver reduced local anesthetic clearance [29].

Our PBPK models successfully predicted ropivacaine pharmacokinetics after EOI plane block. The PBPK model-predicted safe doses of ropivacaine were comparable across age groups and relatively high. This suggests potential safety of EOI plane block across all age groups. However, these predictions should be interpreted cautiously.

Ropivacaine consistently exhibits linear pharmacokinetic, even at high doses (even exceeding 300 mg) [30]. A previous study showed PBPK models allowed for dose extrapolation to three-fold or higher in special populations [31]. However, all simulations were extrapolated from a model developed and calibrated using data from a single clinical dose of 112.5 mg. While PBPK models allow for mechanistic extrapolation, predictions at substantially higher doses (e.g., near or above 300 mg) remain hypothetical and require cautious interpretation. These findings thus represent a preliminary, model-informed exploration rather than clinically validated recommendations. Notably, the recommended doses above 300 mg are derived from model-based simulations and should not be used in clinical practice without direct dose-ranging study validation. Further validation in well-controlled, dose-ranging clinical studies is necessary before any translational application.

Sensitivity analysis suggested that patients with less EOI plane adipose and high EOI plane blood flow (mainly from intercostal artery) may tend to present a higher C_max_, AUC, and shorter T_max_. Consequently, these patients may require a reduced dosage. Similarly, a smaller EOI plane volume may increase C_max_, suggesting that patients with a narrow ribcage may require dose reduction. Furthermore, PBPK sensitivity analysis showed that liver volume exerted less impact on CL than enzyme activity. This aligns with the fact that ropivacaine clearance mainly depends on hepatic enzyme activity and plasma protein binding, due to its low hepatic extraction ratio [32]. Liver cirrhosis significantly impairs hepatic enzyme activity [26]. Accordingly, patients with cirrhosis should receive reduced doses and be monitored closely, as previously reported [26].

Our study has several limitations. First, although the free ropivacaine concentration is more predictive of LAST than total ropivacaine concentration, measurement is far more complex. A previous study reported that the minimum total arterial concentration of ropivacaine in patients with toxic symptoms was 3.4 mg/L [18]. In addition, many studies regard 3.4 mg/L as the total ropivacaine toxic arterial plasma threshold [20]. Thus, due to the feasibility of the assay, we tested the total arterial concentration of ropivacaine. Second, ropivacaine exhibited high protein binding (94%) to AAG, and the unbound fraction is therefore affected by the AAG level. However, we did not measure AAG or evaluate its relationship with ropivacaine for two reasons. Previous research showed that during open liver surgery, AAG levels increase and unbound ropivacaine concentration reduces, suggesting increased tolerance to toxicity [24]. In addition, accurate quantification of AAG was not feasible due to sample degradation from repeated freeze-thaw cycles. Third, the sampling schedule focused on the early absorption phase, with limited coverage of the terminal elimination phase. Thus, it failed to reveal the whole long-term metabolic phase. Our most interested parameter is C_max_, as LAST is primarily associated with it. Additionally, sampling time points extended to approximately half-life. Hence, the data still provided critical pharmacokinetic information to guide clinical practice. Last, gender has minimal impact on ropivacaine pharmacokinetic parameters. We therefore did not establish gender-stratified population simulations for the PBPK model. This PBPK model was developed specifically for Asian populations aged 18–80 years (Child-Pugh grade A and ≤3 liver segments resected) based on available clinical data. Currently, no evidence supports ethnic differences in ropivacaine metabolism. Notably, there is a gap in pharmacokinetic studies on ropivacaine for EOI plane block in patients undergoing liver resection. The lack of comparable data precludes external validation of our model and conclusions, limiting assessment of predictive robustness. Moreover, physiological data on the EOI plane fascial compartment are limited. To ensure dosing safety, we set the EOI plane volume equal to the injection volume (minimum value) and referenced the maximum value reported in the literature. We also did not account for intraoperative changes in AAG, which may affect f_up_ and introduce bias in predictions. Despite these limitations, our study provides novel evidence and validated PBPK models. These results improve the understanding of pharmacokinetics of ropivacaine and its metabolites after EOI plane block in patients undergoing liver resection.

## 4. Materials and Methods

### 4.1. Study Design and Ethics Statement

This study was an observational clinical pharmacokinetic sub-study the used samples derived from a previously published clinical trial protocol [33]. The present analysis focuses solely on pharmacokinetic characterization and PBPK modeling, which is distinct from the parent trial’s primary objectives. The clinical trial was approved by the Institutional Review Board of Peking Union Medical College Hospital (No. I-22PJ747) on 13 October 2022 and was registered on the Chinese Clinical Trial Register website at http://www.chictr.org.cn (ChiCTR2200065745; date of registration: 14 November 2022) before patient enrolment. All patients provided written informed consent, and all procedures complied with the principles of the Declaration of Helsinki. Patients were recruited from a tertiary care hospital. The pharmacokinetic analysis was conducted and reported in line with the ClinPK Statement [34] (Supplementary File S3).

The detailed participants inclusion and exclusion criteria were stated in previous protocol [33]. Briefly, included participants were adults undergoing open liver resection, and the exclusion criteria included any contraindication to EOI plane block, severe systemic disease, or inability to undergo the procedure.

### 4.2. Ultrasound-Guided EOI Plane Block and Sample Collection

The ultrasound-guided EOI plane block was performed before surgery according to a previously described technique [35]. The dose of 112.5 mg (0.375% 30 mL) ropivacaine was administered between the external oblique and intercostal muscles at the right side of the T6/7 intercostal space. No other local anesthetics or medications known to potentially interacting with ropivacaine (according to FDA instruction) were administered [36]. Approximately 30 min after EOI plane block, patients received general anesthesia and surgery.

Arterial blood samples were collected at 5, 10, 30, 60, 120, and 180 min after EOI plane block. Plasma samples were labeled and stored at −80 °C until analysis.

### 4.3. Sample Processing and Analysis

An aliquot of 50 µL plasma was transferred to a 1.5 mL centrifuge tube and spiked with 400 µL acetonitrile, containing 1% formic acid and 50 ng/mL internal standard (IS) for protein precipitation. The entire mixture was then transferred to an Ostro 96-well protein and phospholipid removal plate. Positive pressure was applied for 5 min using a solid-phase extraction device (SaipuRuisi Technology Co., Ltd., Beijing, China). The filtrate was collected and transferred to sample vials for analysis.

UHPLC-MS/MS analysis was conducted using an Agilent 1290 UHPLC (Agilent, Santa Clara, CA, USA) with a 6470 triple quadrupole MS system (UHPLC-QqQ-MS, Agilent, Santa Clara, CA, USA) and performed in multiple reaction monitoring (MRM) mode.

Separation was employed using an ACQUITY UPLC BEH C18 column (2.1 mm × 100 mm, 1.7 µm; Waters, Milford, MA, USA). Mobile phase A consisted of 0.05% (*v*/*v*) formic acid in water, and mobile phase B was 0.05% (*v*/*v*) formic acid in acetonitrile. The injection volume was 2 μL. The gradient program was as follows: 0–2 min, 95% A; 2–4 min, 95–70% A; 4–6 min, 70–60% A. The total run time was 7 min. The column temperature was 35 °C.

Mass spectrometry conditions were as follows: electrospray ionization in positive mode was used. Sheath gas temperature was set at 250 °C, with a sheath gas flow rate of 12 L/min. Drying gas temperature was set at 300 °C, with a drying gas flow rate of 5 L/min. Nebulizer pressure was 40 psi, capillary voltage was 3500 V, and nozzle voltage was 500 V. Optimized ionization parameters are summarized in Table S2 (Supplementary File S2), and detailed procedures are provided in the Supplementary Methods (Supplementary File S1). Method validation was performed in accordance with the US Food and Drug Administration bioanalytical guidance. The chromatograms of ropivacaine and its metabolites are shown in Figure S3 (Supplementary File S2).

Calibrated regression curves showed a good linear relationship ranging from 50 to 10,000 ng/mL for ropivacaine, 5 to 1000 ng/mL for 3-OH ropivacaine and 10 to 2000 ng/mL for PPX. The correlation coefficients (r^2^) of the all three compounds were larger than 0.99, as shown in Figure S4 (Supplementary File S2). The carry-over of ropivacaine, 3-OH ropivacaine, PPX, and the IS were 17.41%, 18.59%, 19.39%, and 0.05%, respectively. No interference peaks from endogenous substances were detected at the corresponding retention times, and no mutual interference was observed between the analytes and the IS, as shown in Table S3 (Supplementary File S2). The intra-batch and inter-batch accuracy of quality control (QC) samples (high quality control (HQC), medium quality control (MQC), low quality control (LQC)) were within 87.0–110.5% and the relative standard deviation (RSD) were all within 15%, as shown in Table S4 (Supplementary File S2).

The RSD for the extraction recovery of QC samples (HQC, MQC, LQC) and IS was all below 15% (Table S5 in Supplementary File S2). The matrix effects of all QC levels were between 87.30% and 106.05% for all tested compounds (Table S6 in Supplementary File S2).

The accuracy of all QC samples in different conditions (at room temperature (20 °C) for 2 and 4 h, at autosampler temperature (4 °C) for 48 h, and after 3 freeze-thaw cycles) was within 15% (Table S7, Supplementary File S2), which met the US Food and Drug Administration guidelines for bioanalytical method validation.

### 4.4. PBPK Development and Validation

PK-Sim^@^ and MoBi^@^ (http://www.open-systems-pharmacology.org) were used to develop the ropivacaine PBPK model. Human pharmaceutical and physicochemical parameters were obtained from the DrugBank database and previously published reports [37], as shown in Table 6. Ropivacaine exhibits linear pharmacokinetics [5] and is mainly metabolized in the liver [6]. K_m_ and V_max_ were used to describe hepatic metabolism, while renal elimination was characterized using renal plasma clearance [36,38].

During EOI plane block, local anesthetic was administered in the fascial plane between the external oblique muscle and intercostal muscle. The fascial space was filled with matrix and adipocytes, through which blood vessels and nerves traverse [39]. The drug diffused into the adipose tissue compartment in the matrix and was distributed or passively diffused into the blood vessels for absorption. According to the anatomy, the administration site (EOI space) was divided into three compartments, including matrix compartment, adipose compartment, and blood compartment. The administration site of EOI plane block was a matrix (Figure S5, Supplementary File S2).

Physiological parameters for the EOI plane space were derived from the external oblique muscle (L6 to L10). The administration site volume was set equal to the injected volume (30 mL). The adipose compartment volume was estimated based on age-dependent adipose proportions in the external oblique muscle: 4.4% (18–45 years), 6.6% (46–59 years), and 10.3% (≥60 years) [40]. The intercostal artery is the primary blood supply of the external abdominal and intercostal muscle [41]. Therefore, the blood flow in the EOI space was assumed to equal intercostal arterial flow (3.48 mL/min/100 g) [42]. The blood compartment volume was assumed to be equal to the adipose tissue volume. The K_f,b_ was 0.3 [43].

The fractional liver volume was adjusted according to the volume of the liver resection in the model [17], as a previous study described. Liver enzyme parameters remained unchanged, as previous studies reported no alteration in CYP3A4 activity following major hepatectomy during the early postoperative period [44]. The physiological parameters of elder population use scaling factors in the software and clinical data. Because CYP3A and CYP1A2 activities have been reported to remain unchanged with aging [45], enzyme activity parameters were not modified

To account for age-related differences in EOI plane adipose tissue, three age cohorts (18–45, 45–59, and 60–79 years) were constructed. Each virtual population consisted of 100 individuals, with the residual liver volume set to 80% based on clinical data (mean residual liver volume of 80% in the study cohort). Key model parameters were derived from our patient cohort to ensure consistency between simulated and observed populations.

The accuracy of the model was validated using clinical data, and by comparing the fold errors of the estimated pharmacokinetic parameters with clinical data. The fold error of the pharmacokinetic parameters was calculated using Equation (1).

(1) $$\mathrm{Fold}\;\mathrm{error}=\frac{\mathrm{Predicted}}{\mathrm{Observed}}$$

The model predictions were considered acceptable if the fold error was between 0.5 and 2. The AFE and the AAFE of all concentration-time data points assess the precision of the model, as shown in Equations (2) and (3) [19].

(2) $$\mathrm{AFE}=10^{\frac{1}{n}\sum \log\left(\frac{\mathrm{Predicted}}{\mathrm{Observed}}\right)}$$

(3) $$\mathrm{AAFE}=10^{\frac{1}{n}\sum \left|\log\left(\frac{\mathrm{Predicted}}{\mathrm{Observed}}\right)\right|}$$

Sensitivity analysis was performed to evaluate the impact of physiological parameters on model output. Varied parameters included liver blood flow, liver volume, CYP enzyme activity, EOI plane volume, EOI plane blood flow, adipose volume, K_f,b_, and f_up_.

Sensitivity analysis was performed by PK-Sim using five sequential steps with a 10% variation range for each parameter. The sensitivity of a pharmacokinetic parameter (PK_j_) to an input parameter (p_i_) was calculated as the ratio of their relative changes, which were determined by perturbing pi by a small amount (Δp_i_) and computing the resultant change in PK_j_ (ΔPK_j_) through comparative simulation, as shown in Equation (4).

(4) $$\mathrm{Si},j=(\frac{\Delta \mathrm{PKj}}{\Delta \;\mathrm{pi}})\cdot (\frac{\mathrm{pi}}{\mathrm{PKj}})$$

The sensitivity of −1.0 implies that a 10% increase of the parameters leads to a 10% decrease of the pharmacokinetics parameter value, and a sensitivity of +0.5 implies that a 10% increase of the parameters leads to a 5% increase of the pharmacokinetics parameter value.

### 4.5. Statistics

According to the trial protocol [33], 37 participants were planned to be enrolled. Ultimately, blood samples were collected from 35 participants for analysis.

DAS 3.0 was used to calculate the pharmacokinetic parameters of ropivacaine, including C_max_, T_max_, AUC, CL, half-life, and apparent volume of distribution (V_d_). All statistical analyses were performed using IBM SPSS Statistics 27.0. Data distribution was evaluated by the Shapiro–Wilk test. The normal distribution data were presented as mean (SD), and the non-normal distribution data were presented as medians (IQR). For categorical variable, data were presented as number and percentage. The Mann–Whitney U test was used to compare non-normally distributed continuous variables. Spearman’s correlation analysis was used to assess relationships between pharmacokinetic parameters and age, weight, height, BMI, hemoglobin, and red blood cell count. Multiple linear regression analysis was applied to explore the associations between pharmacokinetic parameters and the number of resected liver segments, and hepatic inflow occlusion duration, adjusting for confounding factors (age, sex, weight). Because six hypotheses were tested, Bonferroni correction was applied, resulting in a significance threshold of *p* = 0.008. All tests were two-tailed.

## 5. Conclusions

The study described the pharmacokinetics of ropivacaine in patients undergoing liver resection after a single-shot EOI plane block. The dose of 112.5 mg (0.375% 30 mL) ropivacaine is safe and feasible, as plasma concentrations did not exceed the toxic threshold. Patients with less EOI plane adipose tissue, higher EOI plane blood flow, or a narrow ribcage may require dosage reduction. This study also proposed an optimal ropivacaine dosing regimen. Overall, these findings provide valuable clinical guidance to reduce the risk of LAST.

## Figures and Tables

**Table 1:** Participant characteristics.

Characteristic	Value
Male, *n* (%)	17 (60.7%)
Age (year), mean (SD)	59.68 (13.21)
Weight (kg), mean (SD)	64.98 (11.62)
Height (cm), mean (SD)	163.44 (7.56)
BMI (kg/m^2^), mean (SD)	24.24 (3.54)
Age-adjusted Charlson Comorbidity Index, median (IQR)	5 (4–7)
Child-Pugh grade	
A	28(100%)
B	0 (0%)
C	0 (0%)
Surgery duration(h), mean (SD)	3.32 (1.18)
Hemoglobin(g/L), mean (SD)	133.81 (16.14)
Preoperative albumin(g/L), mean (SD)	42.44 (3.38)
Number of hepatic segments resected, mean	1.89 (0.93)
0	3 (10.71%)
1	5 (17.86%)
2	12 (42.86%)
3	8 (28.57%)
Hepatic blood flow occlusion duration (min), mean (SD)	14.14 (18.87)
ASA physical status, *n* (%)	
I	0 (0%)
II	24 (85.7%)
III	4 (14.3%)
IV	0 (0%)

BMI, body mass index; ASA, American Society of Anesthesiologists. For continuous variable, the normal distribution data were presented as mean (SD), and the non-normal distribution data were presented as medians (IQR). For categorical variable, data were presented as number and percentage.

**Table 2:** Pharmacokinetic parameters of ropivacaine and metabolite.

Pharmacokinetic Parameters	Ropivacaine (*n* = 28)	3-OH Ropivacaine (*n* = 25)	PPX (*n* = 14)
C_max_ (ng/mL), median (IQR)	792.56 (625.17–1099.18)	11.37 (7.82–17.15)	33.78 (27.38–48.89)
T_max_ (min), median (IQR)	10 (7.25–10)	30 (30–60)	180 (172.5–180)
AUC (0–∞) (mg/L·min), median (IQR)	152.35 (119.99–219.92)	3.86 (2.92–6.21)	9.37 (7.67–11.08)
Half-time(min), mean, median (IQR)	169.66 (103.89–246.70)	192.60 (112.41–329.95)	166.85 (88.31–180.00)
CL (L/min), mean (SD)	0.78 (0.40)	32.41 (22.41)	11.80 (3.92)
V_d_ (L), median (IQR)	167.90 (130.68–235.96)	10,696.90 (6175.39–13,473.38)	2200.75 (1446.07–2675.46)

C_max_: peak concentration, T_max_: time to C_max_, AUC: area under the curve, CL: clearance, half-life, V_d_: apparent volume of distribution.

**Table 3:** Factors affecting ropivacaine pharmacokinetic parameters.

PK Parameters	Cmax	Tmax	AUC	Half-Life	CL	Vd
Continuous variables (Spearman’s r [95%CI], *p* value)						
Age	0.170 [−0.228, 0.519], *p* = 0.387	−0.024 [−0.403, 0.362], *p* = 0.903	−0.182 [−0.529, 0.216], *p* = 0.353	−0.327 [−0.631, 0.064] *p* = 0.090	0.182 [−0.216, 0.529] *p* = 0.353	−0.055 [−0.429, 0.335], *p* = 0.780
Weight	−0.009 [−0.398, 0.383], *p* = 0.965	0.050 [−0.406, 0.353] *p* = 0.805	−0.144 [−0.410, 0.370], *p* = 0.472	0.204 [−0.202, 0.550], *p* = 0.308	0.024 [−0.370, 0.410], *p* = 0.906	0.144 [−0.260, 0.506], *p* = 0.472
Height	−0.051 [−0.432, 0.346], *p* = 0.802	0.053 [−0.344, 0.434] *p* = 0.792	−0.006 [−0.395, 0.385], *p* = 0.978	0.309 [−0.092, 0.624], *p* = 0.117	−0.221 [−0.563,0.185], *p* = 0.267	0.006 [−0.385, 0.395], *p* = 0.978
BMI	0.085 [−0.315, 0.460], *p* = 0.673	0.075 [−0.325, 0.452], *p* = 0.710	−0.127 [−0.492, 0.277], *p* = 0.529	0.111 [−0.291, 0.481], *p* = 0.580	0.127 [−0.277, 0.492], *p* = 0.529	0.209 [−0.197, 0.554], *p* = 0.296
Hemoglobin	−0.317 [−0.629, 0.084], *p* = 0.107	−0.025 [−0.369, 0.441], *p* = 0.901	−0.498 [−0.712, 0.067], *p* = 0.020	−0.072 [−0.450, 0.327], *p* = 0.720	0.445 [0.067, 0.712], *p* = 0.020	0.244 [−0.161, 0.579], *p* = 0.220
Preoperative Albumin	−0.322 [−0.633, 0.078], *p* = 0.101	−0.377 [−0.664, 0.007], *p* = 0.048	−0.346 [−0.649, 0.051] *p* = 0.077	0.098 [−0.303, 0.470], *p* = 0.625	0.356 [−0.051, 0.649], *p* = 0.063	0.488 [0.124, 0.739], *p* = 0.009
Continuous variables (Multiple regression, β, [95%CI], *p* value)						
Time of hepatic obstruction	0.101 [−7.169, 12.362], *p* = 0.588	0.203 [−0.241, 0.700], *p* = 0.324	0.237 [−1477.701, 5771.674], *p* = 0.233	0.166 [−1.988, 4.662], *p* = 0.415	−0.132 [−0.015, −0.001], *p* = 0.020	0.206 [−2.608, 1.335], *p* = 0.512
Number of liver segments resection	0.237 [−89.221, 297.633], *p* = 0.277	0.027 [−4.169, 14.711], *p* = 0.260	0.003 [−27.236, 126.416], *p* = 0.196	−0.087 [−67.552, 68.577], *p* = 0.998	−0.048 [−0.183, 0.115], *p* = 0.642	0.105 [−97.947, 78.296], *p* = 0.820
Categorical variables (Mann-Whitney U test, *p* value)						
Sex	0.433	0.422	0.285	0.84	0.236	0.937

PK: pharmacokinetic, 95%CI: confidence interval, β: Standardized regression coefficient in multiple regression (adjusted for age, sex and weight), body mass index (BMI), C_max_: peak concentration, T_max_: time to C_max_, AUC: area under the curve, CL: clearance, V_d_: apparent volume of distribution. After Bonferroni correction, *p* < 0.008 is statistically significant.

**Table 4:** Predicted C_max_ of ropivacaine following dose adjustment in different age.

Age	Scenario	C_max_ (ng/mL)
18–45	335 mg single	3397.0
	336 mg single	3407.1
46–60	333 mg single	3392.2
	334 mg single	3402.4
60–79	310 mg single	3396.2
	311 mg single	3407.2

C_max_: peak concentration.

**Table 5:** Summary table.

Key novel findings	C_max_ of total ropivacaine after single shot of ultrasound guided EOI plane block with 30 mL of 0.375% ropivacaine is below systemic toxicity threshold. Ropivacaine after EOI plane block has rapid absorption (T_max_: 10 min) Ropivacaine after EOI plane block has low risk of metabolite accumulation (the fraction of ropivacaine metabolized to 3-OH ropivacaine and PPX is 2.71% and 7.42%) No more than 3 liver segments or rapid hepatic obstruction (mean 14.14 min) exerts minimal effect on ropivacaine clearance The patients with less EOI plane adipose and high EOI plane blood flow (mainly from intercostal artery) may tend to present a higher C_max_, AUC, and shorter T_max_.
Clinical implications	Indicate the safety of single shot of ropivacaine administration in EOI plane for patients with open liver resection with respect to the risk of systemic toxicity. Suggest that post-block monitoring for 10–30 min is appropriate For single shot of EOI plane block, monitoring of metabolite concentration is not necessary No dosage adjustment for ropivacaine is required when ≤3 hepatic segments are resected Patients with less EOI plane adipose and high EOI plane blood flow (mainly from intercostal artery) may require a reduced dosage.
Study limitations	The unbound concentration of ropivacaine was not measured. The AAG concentration was not measured. The sampling time points not fully cover ropivacaine’s prolonged metabolic phase The PBPK model lacks external validation

**Table 6:** Parameters used to establish the physiology-based pharmacokinetic model for ropivacaine.

Parameters	Value	Source
Molecular mass (g/mol)	274.4	Drugbank [38]
LogP	2.9	Drugbank [38]
*f*_up_	0.06	Drugbank [38]
pKA	Acid 7.82, Base 13.62	Drugbank [38]
Solubility (mg/mL)	53.8	Drugbank [38]
V_max,CYP1A2_ (pmol/min /mg protein)	46	Ekström G, et al. [37]
K_m,CYP1A2_ (µmol/L)	16	Ekström G, et al. [37]
Renal clearance (mL/min)	1	FDA [36]

K_m_, Michaelis–Menten constant; V_max_, maximum reaction velocity; CYP, cytochrome P450 enzymes.

## Data Availability

The original contributions presented in this study are included in the article/Supplementary Materials. Further inquiries can be directed to the corresponding authors.

## Supplementary Materials

The supporting information can be downloaded at: https://www.mdpi.com/article/10.3390/ph19030348/s1, Supplementary File S1: Supplementary method. Supplementary File S2: Figure S1: Sample inclusion and exclusion flowchart. 3-OH ropivacaine: 3-hydroxy-ropivacaine; PPX: 2′,6′-pipecoloxylidide; LLOQ; quantitative lower limit. Figure S2: PBPK model sensitivity analysis of the related parameters and their effects on pharmacokinetic data (The sensitivities are dimensionless quantities). A, B, C, D is effects on clearance (CL), area under curve (AUC), peak concentration (Cmax), time to peak concentration (Tmax), respectively. As an example, a sensitivity of −1.0 implies that a 10% increase of the parameters leads to a 10% decrease of the pharmacokinetic parameter value, and a sensitivity of +0.5 implies that a 10% increase of the parameters leads to a 5% increase of the pharmacokinetic parameter value. EOI, external oblique intercostal. Figure S3: Chromatograms of ropivacaine and its metabolites. Mobile phase A = 0.05% formic acid water, mobile phase B = 0.05% formic acid acetonitrile. 1 is 3-OH ropivacaine, 2 is ropivacaine, 3 is PPX, respectively. 3-OH ropivacaine: 3-hydroxy-ropivacaine; PPX, 2′,6′-pipecoloxylidide. Figure S4: Linear regression plot for ropivacaine, 3-OH ropivacaine and PPX. 3-OH ropivacaine: 3-hydroxy-ropivacaine; PPX: 2′,6′-pipecoloxylidide. Figure S5: Structure of the PBPK model developed in this paper. The white compartment is the structure of the adult population. Light grey compartments are EOI block dosing structures. Black solid arrows indicate drug transport through the bloodstream. Dashed arrows indicate drug transport through gastrointestinal motility or excretion through the bile. EOI: external oblique intercostal. Table S1: Comparison of mean predicted and observed pharmacokinetic parameters. Table S2 Ropivacaine and its metabolites multiple reaction monitoring parameters. Table S3: Interference of blank matrix on analytes and interior standard. Table S4: Precision and accuracy of ropivacaine, 3-OH ropivacaine and PPX in plasma. Table S5: Extract recovery relative standard deviation. Table S6: Matrix effect factor of ropivacaine, 3-OH ropivacaine and PPX in human plasma. Table S7: Summary of stability in different matrices and storage conditions (*n* = 3). Supplementary File S3: ClinPK checklist.

## References

[1] Xu Y., Ye M., Liu F., Hong Y., Kang Y., Li Y., Li H., Xiao X., Yu F., Zhou M. (2023). Efficacy of prolonged intravenous lidocaine infusion for postoperative movement-evoked pain following hepatectomy: A double-blinded, randomised, placebo-controlled trial. Br. J. Anaesth..

[2] Kaye A.D., Chernobylsky D.J., Thakur P., Siddaiah H., Kaye R.J., Eng L.K., Harbell M.W., Lajaunie J., Cornett E.M. (2020). Dexmedetomidine in Enhanced Recovery After Surgery (ERAS) Protocols for Postoperative Pain. Curr. Pain Headache Rep..

[3] Doymus O., Ahiskalioglu A., Kaciroglu A., Bedir Z., Tayar S., Yeni M., Karadeniz E. (2024). External Oblique Intercostal Plane Block Versus Port-Site Infiltration for Laparoscopic Sleeve Gastrectomy: A Randomized Controlled Study. Obes. Surg..

[4] Liotiri D., Diamantis A., Papapetrou E., Grapsidi V., Sioka E., Stamatiou G., Zacharoulis D. (2023). External oblique intercostal (EOI) block for enhanced recovery after liver surgery: A case series. Anaesth. Rep..

[5] Wyatt K.E., Liu C.J., Moffett B., Vogel A.M., Medellin E., Owens-Stuberfield M., Lee A., Baijal R.G. (2022). Ropivacaine concentrations after single-shot erector spinae plane block in children: A pilot pharmacokinetic modelling study. Br. J. Anaesth..

[6] Pere P.J., Ekstrand A., Salonen M., Honkanen E., Sjovall J., Henriksson J., Rosenberg P.H. (2011). Pharmacokinetics of ropivacaine in patients with chronic renal failure. Br. J. Anaesth..

[7] Rahiri J., Tuhoe J., Svirskis D., Lightfoot N.J., Lirk P.B., Hill A.G. (2017). Systematic review of the systemic concentrations of local anaesthetic after transversus abdominis plane block and rectus sheath block. Br. J. Anaesth..

[8] Riff C., Le Caloch A., Dupouey J., Allanioux L., Leone M., Blin O., Bourgoin A., Guilhaumou R. (2022). Local Anesthetic Plasma Concentrations as a Valuable Tool to Confirm the Diagnosis of Local Anesthetic Systemic Toxicity? A Report of 10 Years of Experience. Pharmaceutics.

[9] Kumar S.K., Rao V., Morris R.G., Watts R.W., Westley I.S. (2014). Ropivacaine (total and unbound) and AGP concentrations after transversus abdominis plane block for analgesia after abdominal surgery. Ther. Drug. Monit..

[10] Calenda E., Baste J.M., Hajjej R., Danielou E., Peillon C. (2014). Toxic plasma concentration of ropivacaine after a paravertebral block in a patient suffering from severe hypoalbuminemia. J. Clin. Anesth..

[11] Schwenk E.S., Lam E., Abulfathi A.A., Schmidt S., Gebhart A., Witzeling S.D., Mohamod D., Sarna R.R., Roy A.B., Zhao J.L. (2023). Population pharmacokinetic and safety analysis of ropivacaine used for erector spinae plane blocks. Reg. Anesth. Pain Med..

[12] Liu T., Yang J., Wang Y., Jiang W., Luo Y., Feng X., Mei W. (2023). Interfascial plane block: A new anesthetic technique. Anesthesiol. Perioper. Sci..

[13] Fettiplace M.R., Weinberg G., Chiang C., Nixon H.C., Gitman M. (2025). Contemporary local anaesthetic-associated adverse events and mortality: A pharmacovigilance analysis of a US reporting system. Br. J. Anaesth..

[14] Ren H.C., Zhu X., Hao K., Le S.Y. (2025). Editorial: Application of PKPD modeling in drug discovery and development. Front. Pharmacol..

[15] Liu X., Wang W., Chen J., Chen D., Tao Y., Ouyang D. (2024). PBPK/PD Modeling of Nifedipine for Precision Medicine in Pregnant Women: Enhancing Clinical Decision-Making for Optimal Drug Therapy. Pharm. Res..

[16] Lee S.M., Jang J.H., Jeong S.H. (2025). Precision Pharmacokinetics of Quetiapine: A Physiologically Based Model Incorporating Liver Cirrhosis and CYP3A4 Polymorphisms. J. Clin. Pharmacol..

[17] Koller A., Grzegorzewski J., Tautenhahn H.M., Konig M. (2021). Prediction of Survival After Partial Hepatectomy Using a Physiologically Based Pharmacokinetic Model of Indocyanine Green Liver Function Tests. Front. Physiol..

[18] Knudsen K., Beckman Suurkula M., Blomberg S., Sjovall J., Edvardsson N. (1997). Central nervous and cardiovascular effects of i.v. infusions of ropivacaine, bupivacaine and placebo in volunteers. Br. J. Anaesth..

[19] Poulin P., Jones R.D., Jones H.M., Gibson C.R., Rowland M., Chien J.Y., Ring B.J., Adkison K.K., Ku M.S., He H. (2011). PHRMA CPCDC initiative on predictive models of human pharmacokinetics, part 5: Prediction of plasma concentration-time profiles in human by using the physiologically-based pharmacokinetic modeling approach. J. Pharm. Sci..

[20] Ling J., Xu C., Tang L., Qiu L., Hu N. (2025). Comparison of the pharmacokinetic variations of different concentrations of ropivacaine used for serratus anterior plane block in patients undergoing thoracoscopic lobectomy: A population pharmacokinetics analysis. Front. Pharmacol..

[21] Murouchi T., Iwasaki S., Yamakage M. (2016). Quadratus Lumborum Block: Analgesic Effects and Chronological Ropivacaine Concentrations After Laparoscopic Surgery. Reg. Anesth. Pain Med..

[22] Karmakar M.K., Ho A.M., Law B.K., Wong A.S., Shafer S.L., Gin T. (2005). Arterial and venous pharmacokinetics of ropivacaine with and without epinephrine after thoracic paravertebral block. Anesthesiology.

[23] Behnke H., Worthmann F., Cornelissen J., Kahl M., Wulf H. (2002). Plasma concentration of ropivacaine after intercostal blocks for video-assisted thoracic surgery. Br. J. Anaesth..

[24] Zhang J., Lv H., Shen J., Ai Z., Liu M., Liu X., Liu T., Shen B., Yu H., Yu X. (2025). Altered Pharmacokinetics of Ropivacaine in Patients Undergoing Laparoscopic Major Hepatectomy. Pharmaceutics.

[25] Aarons L., Sadler B., Pitsiu M., Sjovall J., Henriksson J., Molnar V. (2011). Population pharmacokinetic analysis of ropivacaine and its metabolite 2’,6’-pipecoloxylidide from pooled data in neonates, infants, and children. Br. J. Anaesth..

[26] Jokinen M.J., Neuvonen P.J., Lindgren L., Hockerstedt K., Sjovall J., Breuer O., Askemark Y., Ahonen J., Olkkola K.T. (2007). Pharmacokinetics of ropivacaine in patients with chronic end-stage liver disease. Anesthesiology.

[27] Burm A.G., Stienstra R., Brouwer R.P., Emanuelsson B.M., van Kleef J.W. (2000). Epidural infusion of ropivacaine for postoperative analgesia after major orthopedic surgery: Pharmacokinetic evaluation. Anesthesiology.

[28] Butiulca M., Farczadi L., Imre S., Vari C.E., Vlase L., Azamfirei L., Lazar A.E. (2025). Evaluation of Ropivacaine and 3-OH-Ropivacaine Pharmacokinetics Following Interpectoral Nerve Block via LC-MS/MS-A Pilot Study. Int. J. Mol. Sci..

[29] Crouch C.E., Wilkey B.J., Hendrickse A., Kaizer A.M., Schniedewind B., Christians U., Henthorn T.K., Fernandez-Bustamante A. (2023). Lidocaine Intraoperative Infusion Pharmacokinetics during Partial Hepatectomy for Living Liver Donation. Anesthesiology.

[30] Gromov K., Grassin-Delyle S., Foss N.B., Pedersen L.M., Nielsen C.S., Lamy E., Troelsen A., Urien S., Husted H. (2021). Population pharmacokinetics of ropivacaine used for local infiltration anaesthesia during primary total unilateral and simultaneous bilateral knee arthroplasty. Br. J. Anaesth..

[31] Yellepeddi V., Bayless S., Parrot M., Sherwin C.M. (2024). Optimal Dosing Recommendations of Clonidine in Pediatrics Using Physiologically Based Pharmacokinetic Modeling. J. Pediatr. Pharmacol. Ther..

[32] Ollier E., Heritier F., Bonnet C., Hodin S., Beauchesne B., Molliex S., Delavenne X. (2015). Population pharmacokinetic model of free and total ropivacaine after transversus abdominis plane nerve block in patients undergoing liver resection. Br. J. Clin. Pharmacol..

[33] Tang J., Hua Q., Zhang Y., Nie W., Yu S., Zhang J. (2024). Effects of ultrasound-guided external oblique intercostal plane block on the postoperative analgesia after open liver surgery: Study protocol for a randomised controlled trial. Trials.

[34] Kanji S., Hayes M., Ling A., Shamseer L., Chant C., Edwards D.J., Edwards S., Ensom M.H., Foster D.R., Hardy B. (2015). Reporting Guidelines for Clinical Pharmacokinetic Studies: The ClinPK Statement. Clin. Pharmacokinet..

[35] Elsharkawy H., Kolli S., Soliman L.M., Seif J., Drake R.L., Mariano E.R., El-Boghdadly K. (2021). The External Oblique Intercostal Block: Anatomic Evaluation and Case Series. Pain Med..

[36] Administration (2022). USFaD: NAROPIN® (Ropivacaine Hydrochloride) Injection, for Epidural, Perineural, or Infiltration Use. https://www.dailymed.nlm.nih.gov/dailymed/fda/fdaDrugXsl.cfm?setid=23d2d448-a744-4877-9f2d-7e57c198da89&type=display.

[37] Ekstrom G., Gunnarsson U.B. (1996). Ropivacaine, a new amide-type local anesthetic agent, is metabolized by cytochromes P450 1A and 3A in human liver microsomes. Drug Metab. Dispos..

[38] (2025). Online D: DrugBank Online. Ropivacaine (DB00296). https://go.drugbank.com/drugs/DB00296.

[39] Chin K.J., Versyck B., Elsharkawy H., Rojas Gomez M.F., Sala-Blanch X., Reina M.A. (2021). Anatomical basis of fascial plane blocks. Reg. Anesth. Pain Med..

[40] Jourdan A., Soucasse A., Scemama U., Gillion J.F., Chaumoitre K., Masson C., Bege T. (2020). For Club H: Abdominal wall morphometric variability based on computed tomography: Influence of age, gender, and body mass index. Clin. Anat..

[41] Schlenz I., Burggasser G., Kuzbari R., Eichberger H., Gruber H., Holle J. (1999). External oblique abdominal muscle: A new look on its blood supply and innervation. Anat. Rec..

[42] De Bisschop C., Montaudon M., Glenet S., Guenard H. (2017). Feasibility of intercostal blood flow measurement by echo-Doppler technique in healthy subjects. Clin. Physiol. Funct. Imaging.

[43] Feldman H.S., Hartvig P., Wiklund L., Doucette A.M., Antoni G., Gee A., Ulin J., Langstrom B. (1997). Regional distribution of 11C-labeled lidocaine, bupivacaine, and ropivacaine in the heart, lungs, and skeletal muscle of pigs studied with positron emission tomography. Biopharm. Drug Dispos..

[44] Jedamzik J., Muhlbacher J., Fitschek F., Schwarz C., Burhenne J., Asenbaum U., Kaczirek K., Mikus G. (2020). No alteration of Cyp3A4 activity after major hepatectomy in the early postoperative period—A prospective before-after study. Int. J. Surg..

[45] Cui C., Valerie Sia J.E., Tu S., Li X., Dong Z., Yu Z., Yao X., Hatley O., Li H., Liu D. (2021). Development of a physiologically based pharmacokinetic (PBPK) population model for Chinese elderly subjects. Br. J. Clin. Pharmacol..

