# Supplementary material for: Physiology-Based Pharmacokinetic Modeling of Ropivacaine After External Oblique Intercostal Plane Block in Open Liver Surgery Patients
Source: Pharmaceuticals (Basel). 2026 Feb 24;19(3):348. doi: 10.3390/ph19030348 (PMC13029281; doi:10.3390/ph19030348)
Supplement: Supplementary file 1 [file pharmaceuticals-19-00348-s001.zip › Supplementary File S1.pdf]

Supplementary Methods

1 Sample processing and analysis

1.1 Materials and reagents

Ropivacaine (CAS 27262-40-4, purity: 99%), 3-OH ropivacaine (CAS 163589-30-8, purity: 98%), ropivacaine-D7 (CAS 1217667-10-1, chemical purity: 98%) were purchased from the Beijing Bailingwei Technology (Beijing, China). PPX (lot 0559567, purity:98%) was purchased from Shanghai Haohong Biopharmaceutical Technology (Shanghai, China). Formic acid was obtained from Shanghai Aladdin Biochemical Technology (Shanghai, China). HPLC-grade acetonitrile was obtained from Beijing Bailingwei Technology (Beijing, China).

1.2 Instrument settings

UHPLC-MS/MS analysis was conducted using an Agilent 1290 UHPLC with a 6470 triple quadrupole MS system (UHPLC-QqQ-MS), performed in multiple reaction monitoring (MRM) mode.

Separation was employed using an ACQUITY UPLC BEH C18 column (2.1 mm × 100 mm, 1.7 μm; Waters, USA). Mobile phase A consisted of 0.05% (v/v) formic acid in water, and mobile phase B was 0.05% (v/v) formic acid in acetonitrile. The injection volume was 2 μL. The elution gradient was: 0 -2 min, 95% A; 2 -4 min, 95% -70% A; 4 -6 min, 70% -60% A. The total run time was 7 min. The column temperature was 35 °C.

The mass spectrometry conditions were as follows: positive mode electrospray ionization was used, sheath gas temperature, 250 °C; sheath gas flow, 12 L/min; drying gas temperature, 300 °C; drying gas flow, 5 L/min; nebulizer pressure, 40 psi; capillary voltage, 3500 V; and nozzle voltage, 500 V.

1.3 Preparation of calibration standards and quality control samples

Mixed stock solutions of ropivacaine, 3-hydroxy-ropivacaine(3-OH ropivacaine), PPX(2′,6′-pipecoloxylidide) were prepared having concentrations of 50,000 ng/mL, 5,000 ng/mL, 10,000 ng/mL, respectively. These stock solutions were used to obtain the standard solutions. The calibration standards (CS) samples and quality control (QC) samples were prepared by spiking 80 μL blank plasma with 20 μL of the

corresponding mixed working solutions. The final concentration range was 50-10,000 ng/mL for ropivacaine, 5-1,000 ng/mL for 3-OH ropivacaine, 10-2,000 ng/mL for PPX. An internal standard (IS) was ropivacaine-D7 at 50 ng/mL.

Meanwhile, the ropivacaine/3-OH-ropivacaine/PPX concentrations of QC samples were 100/10/20 (low quality control, LQC), 1000/100/200 (medium quality control, MQC) and 8000/800/1600 (high quality control, HQC) ng/mL.

1.4 Sample preparation

An aliquot of 50 μL plasma was transferred to a 1.5 mL centrifuge tube, which was spiked with 400 μL acetonitrile containing 1% formic acid and 50 ng/mL IS for protein precipitation. Transferred all the sample solution to the Ostro 96-well protein & phospholipid removal plate. Positive pressure was applied to the solid-phase extraction device for 5 minutes. Then, collected the filtrate and transferred it into the sample bottle for testing.

2.1 Method validation

2.1.1 Selectivity and carry-over effect

Selectivity was assessed by comparing blank samples (from six donors) with those spiked at the lower limit of quantification (LLOQ); both were processed without the IS. To assess carry-over, a blank sample was analyzed directly after the upper limit of quantification (ULOQ). Regarding the acceptance criteria, the peak areas of blank matrix samples and carry-over samples could not surpass 20% of the LLOQ samples for analytes, and 5% for the IS.

2.1.2. Linearity and calibration model

The standard curves included calibration standards at eight concentrations, with blank plasma excluded. Standard curves were generated from a linear regression model with a weighting factor (1/x) to determine slope, intercept and correlation coefficient (r^2). Acceptance criteria for the calibration curves were the relative error of at least 75% CS samples was within the 15% range, and the LLOQ was allowed a deviation of ≤20%.

2.1.3. Precision and accuracy

Intra-day and inter-day accuracy and precision were evaluated by analyzing six

replicates of the LQC, MQC, and HQC samples. The accuracy was required range from 85% to 115%. The precision, expressed as both intra-day and inter-day relative standard deviation (RSD) of QC samples, had to be below 15%.

2.1.4. Matrix Effect and recovery

The recovery and matrix effect were evaluated in six different lots of blank human plasma at three QC concentration levels (HQC, MQC and LQC). Matrix effect was assessed by comparing the peak area ratios of analytes to IS in spiked blank matrix with those in pure standard solutions. The acceptance criterion was matrix effect factor range from 85% to 115%.

Extraction recovery was defined as the ratio of the peak areas (analyte and IS) in pre-extraction spiked samples to those in post-extraction spiked samples at equivalent concentrations. The RSD of extraction recovery should be less than 15%.

2.1.5. Stability

The stability of three QC concentration levels (HQC, MQC and LQC) under different storage conditions was investigated, with each sample prepared in triplicate. The following conditions were evaluated: at room temperature (20 °C) for 2 and 4 hours; under auto-sampler conditions (4 °C) for 48 hours; after three freeze-thaw cycles (from -80°C to room temperature, with ≥12 hours frozen between cycles). The precision and accuracy should be below 15%.
