# Supplementary material for: Physiology-Based Pharmacokinetic Modeling of Ropivacaine After External Oblique Intercostal Plane Block in Open Liver Surgery Patients
Source: Pharmaceuticals (Basel). 2026 Feb 24;19(3):348. doi: 10.3390/ph19030348 (PMC13029281; doi:10.3390/ph19030348)
Supplement: Supplementary file 1 [file pharmaceuticals-19-00348-s001.zip › Supplementary File S2.pdf]

Supplementary File S2

Table S1 Comparison of mean predicted and observed pharmacokinetic parameters

Pharmacokinetic parameters	Predicted	Observed	Fold-error
C_{max} (ng/mL)	1173.08	792.56	1.48
T_{max} (min)	9.00	10.00	0.90
$AUC_{(0-\infty)}$ (mg/L·min)	164.46	152.35	1.08
CL (L/min)	0.69	0.78	0.88
Half-time (min)	281.68	169.66	1.66
Vd (L)	206.41	167.90	1.23

CL: clearance; AUC: area under curve; C_{max}: peak concentration; T_{max}: time to peak concentration; Vd: apparent volume of distribution.

Table S2 Ropivacaine and its metabolites Multiple Reaction Monitoring parameters

	Fragmentor	CE (eV)	Parent ion (m/z)	Quantitative ions (m/z)	Qualitative ions (m/z)
Ropivacaine	110	20	275.6	126.2	84.4
3-OH ropivacaine	135	25	291.6	126.4	84.4
PPX	110	15	233.5	84.4	56.2
Ropivacaine-D7	135	25	282.6	133.4	85.3

CE: collision energy; 3-OH ropivacaine: 3-hydroxy-ropivacaine; PPX: 2′,6′-pipecoloxylidide

Table S3Interference of blank matrix on analytes and interior standard

Human Plasma	Impurity Peak Area / Peak Area of 3-OH ropivacaine LLOQ	Impurity Peak Area / Peak Area of PPX LLOQ	Impurity Peak Area / Peak Area of Ropivacaine LLOQ	Impurity Peak Area / Peak Area of Internal Standard LLOQ
Source 1	19.6%	19.9%	6.43%	0.01%
Source 2	19.8%	19.6%	6.98%	0.03%
Source 3	19.8%	19.9%	5.77%	0.03%
Source 4	19.7%	19.9%	6.03%	0.02%
Source 5	19.8%	19.8%	6.71%	0.02%
Source 6	19.7%	19.7%	6.09%	0.00%
Average	19.7%	19.8%	6.34%	0.02%

LLOQ: quantitative lower limit; 3-OH ropivacaine: 3-hydroxy-ropivacaine; PPX: 2′,6′-pipecoloxylidide

Table S4. Precision and accuracy of ropivacaine, 3-OH ropivacaine and PPX in plasma

Compounds	QC (ng/mL)	Intra-batch (*n*=3)		Inter-batch (*n*=9)	
		Accuracy	RSD	Accuracy	RSD
Ropivacaine	100	94.15%	1.24%	103.6%	7.90%
	1000	99.78%	0.92%	100.3%	0.82%
	8000	87.04%	0.87%	85.83%	1.22%
3-OH ropivacaine	10	107.4%	3.27%	110.1%	3.52%
	100	90.72%	0.96%	90.62%	1.18%
	800	88.00%	2.79%	87.84%	2.09%
PPX	20	106.1%	1.30%	110.5%	3.50%
	200	104.9%	1.53%	95.33%	8.92%
	1600	88.00%	5.37%	94.99%	3.00%

RSD: relative standard deviation; n: number of replicates; 3-OH ropivacaine: 3-hydroxy-ropivacaine; PPX: 2′,6′-pipecoloxylidide; QC: quality control; RSD: relative standard deviation

Table S5 Extract recovery relative standard deviation

Compounds	Concentration (ng/mL)	RSD
Ropivacaine	100	9.09%
	1000	7.16%
	8000	13.7%
3-OH ropivacaine	10	14.55%
	100	11.4%
	800	6.24%
PPX	20	11.3%
	200	4.81%
	1600	10.1%

RSD: relative standard deviation; 3-OH ropivacaine: 3-hydroxy-ropivacaine; PPX: 2′,6′-pipecoloxylidide

Table S6 Matrix effect factor of ropivacaine, 3-OH ropivacaine and PPX in human plasma

Human plasma lot	Ropivacaine (ng/mL)			3-OH ropivacaine (ng/mL)			PPX (ng/mL)		
	100	1000	8000	10	100	800	20	200	1600
1	104.5%	95.75%	92.73%	101.9%	99.14%	94.47%	102.2%	99.26%	94.47%
2	87.3%	88.40%	91.12%	97.30%	91.87%	94.46%	104.3%	95.02%	94.46%
3	102.1%	88.42%	89.51%	99.10%	93.63%	96.71%	102.9%	95.42%	96.71%
4	106.1%	94.38%	87.33%	95.33%	87.64%	85.26%	99.25%	93.16%	85.26%
5	102.1%	91.31%	92.15%	87.41%	88.25%	86.69%	94.67%	93.60%	86.69%
6	101.0%	99.05%	98.55%	91.34%	86.97%	95.94%	96.66%	99.59%	95.94%

3-OH ropivacaine: 3-hydroxy-ropivacaine; PPX: 2′,6′-pipecoloxylidide

Table S7. Summary of stability in different matrices and storage conditions (n = 3)

Condition	Ropivacaine (ng/mL)			3-OH ropivacaine (ng/mL)			PPX (ng/mL)		
	100	1000	8000	10	100	800	20	200	1600
room temperature (20 °C) 2 h									
Accuracy	113.75%	102.24%	85.35%	108.31%	95.23%	87.15%	104.24%	95.99%	91.48%
RSD	1.15%	2.73%	0.20%	10.94%	2.11%	1.78%	7.96%	6.82%	0.52%
room temperature (20 °C) 4 h									
Accuracy	111.88%	99.96%	85.28%	109.26%	90.80%	85.92%	111.71%	93.46%	89.57%
RSD	0.94%	1.35%	0.45%	6.59%	1.20%	1.06%	2.92%	0.92%	1.20%
autosampler temperature (4 °C) 48 h									
Accuracy	97.66%	97.15%	86.08%	110.19%	91.38%	94.19%	105.65%	98.92%	102.40%
RSD	13.28%	5.18%	0.52%	6.10%	5.02%	0.79%	3.23%	10.16%	4.74%
Freeze-thaw stability (–80 ℃, 3 times)									
Accuracy	97.66%	97.15%	86.08%	110.19%	91.38%	94.19%	105.65%	98.92%	102.40%
RSD	13.28%	5.18%	0.52%	6.10%	5.02%	0.79%	3.23%	10.16%	4.74%

3-OH ropivacaine: 3-hydroxy-ropivacaine; PPX: 2′,6′-pipecoloxylidide; RSD: relative standard deviation

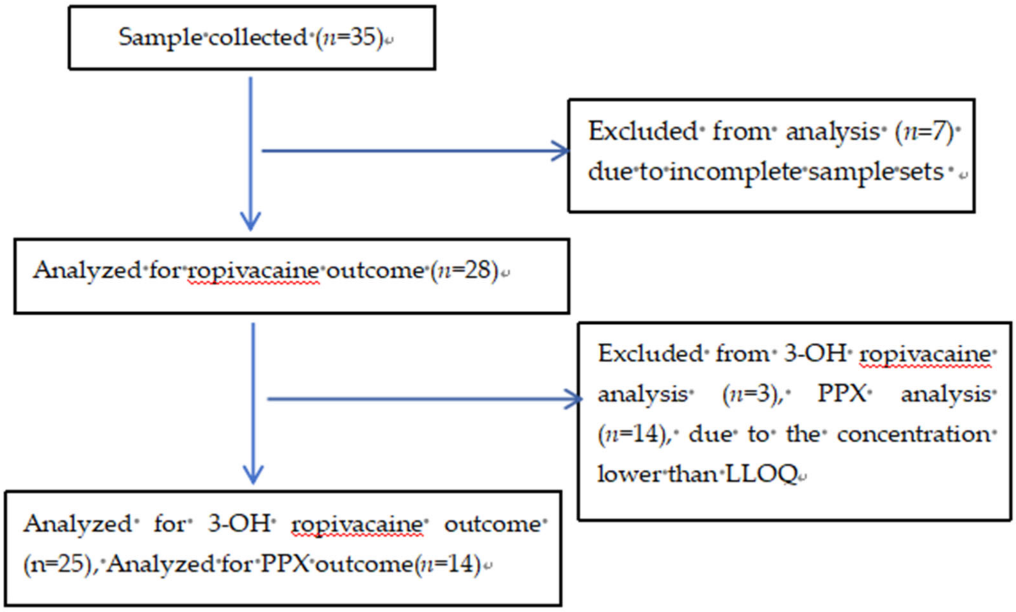

Figure S1 Sample inclusion and exclusion flowchart. 3-OH ropivacaine: 3-hydroxy-ropivacaine; PPX: 2′,6′-pipecoloxylidide; LLOQ; quantitative lower limit.

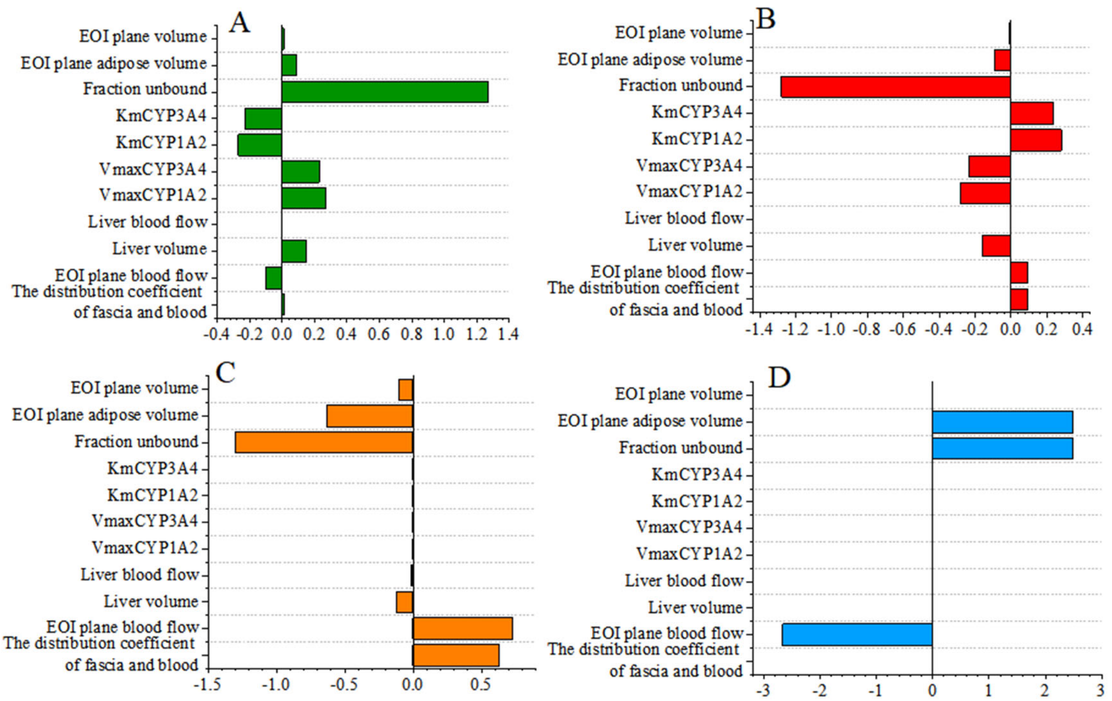

Figure S2 PBPK model sensitivity analysis of the related parameters and their effects on pharmacokinetic data (The sensitivities are dimensionless quantities). A, B, C, D is effects on clearance (CL), area under curve (AUC), peak concentration (C_{max}), time to peak concentration (T_{max}), respectively. As an example, a sensitivity of -1.0 implies that a 10% increase of the parameters leads to a 10% decrease of the pharmacokinetic parameter value, and a sensitivity of +0.5 implies that a 10% increase of the parameters leads to a 5% increase of the pharmacokinetic parameter value. EOI, external oblique intercostal.

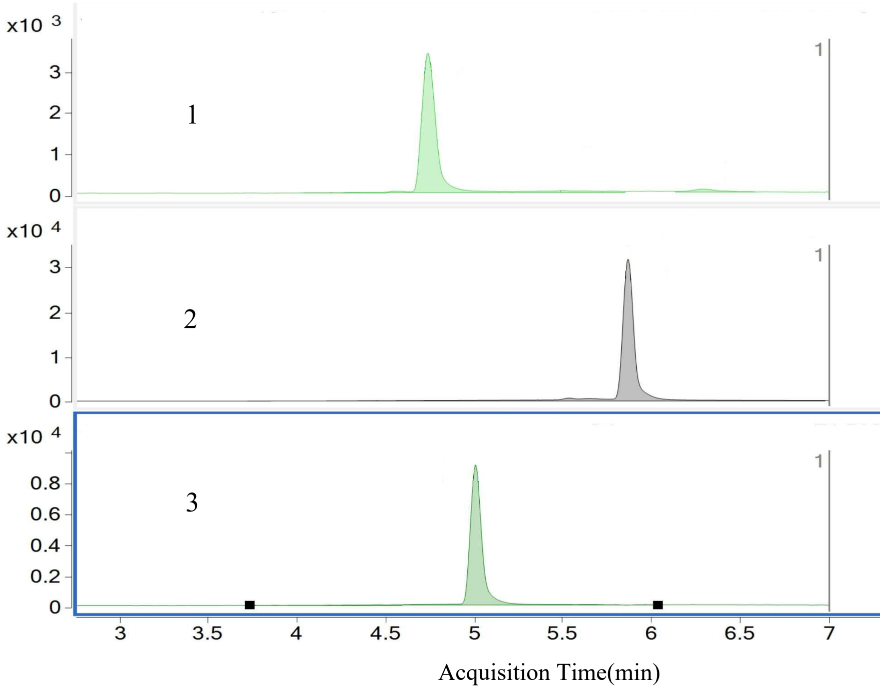

Figure S3 Chromatograms of ropivacaine and its metabolites. Mobile phase A = 0.05% formic acid water, mobile phase B = 0.05% formic acid acetonitrile. 1 is 3-OH ropivacaine, 2 is ropivacaine, 3 is PPX, respectively. 3-OH ropivacaine: 3-hydroxy-ropivacaine; PPX, 2′,6′-pipecoloxylidide.

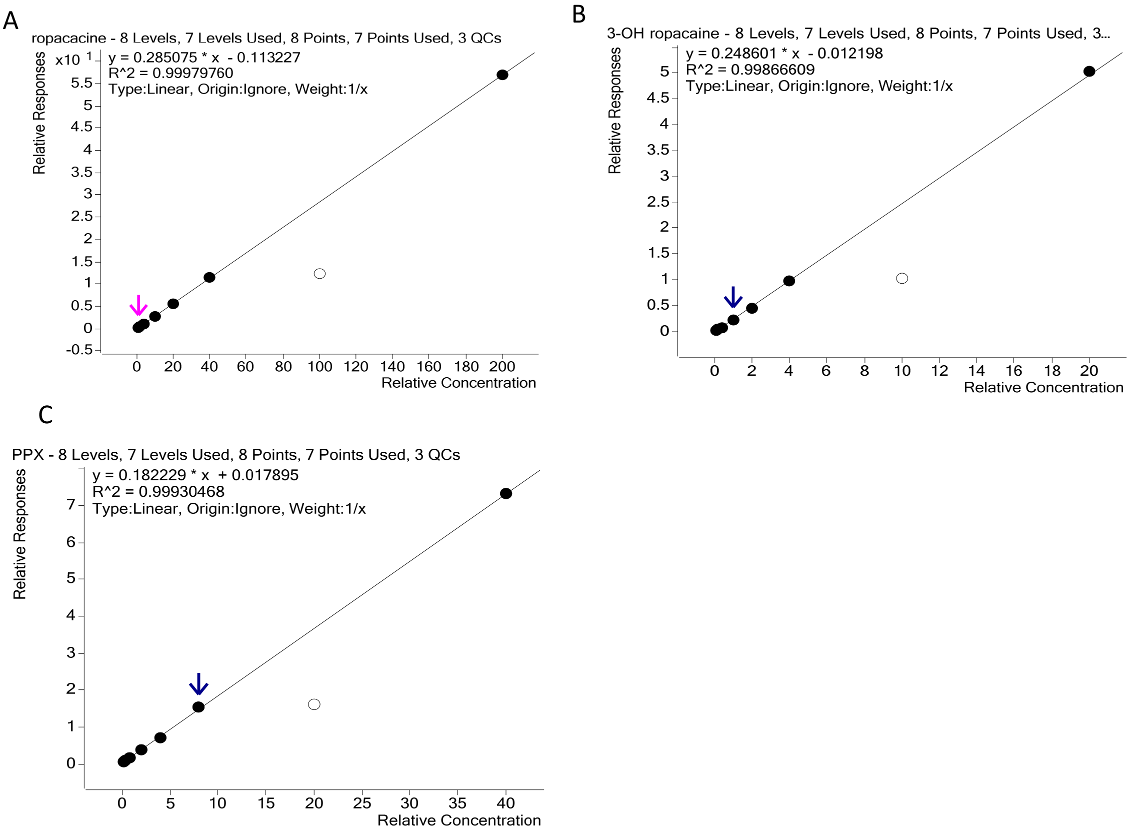

Figure S4 Linear regression plot for ropivacaine, 3-OH ropivacaine and PPX. 3-OH ropivacaine: 3-hydroxy-ropivacaine; PPX: 2′,6′-pipecoloxylidide.

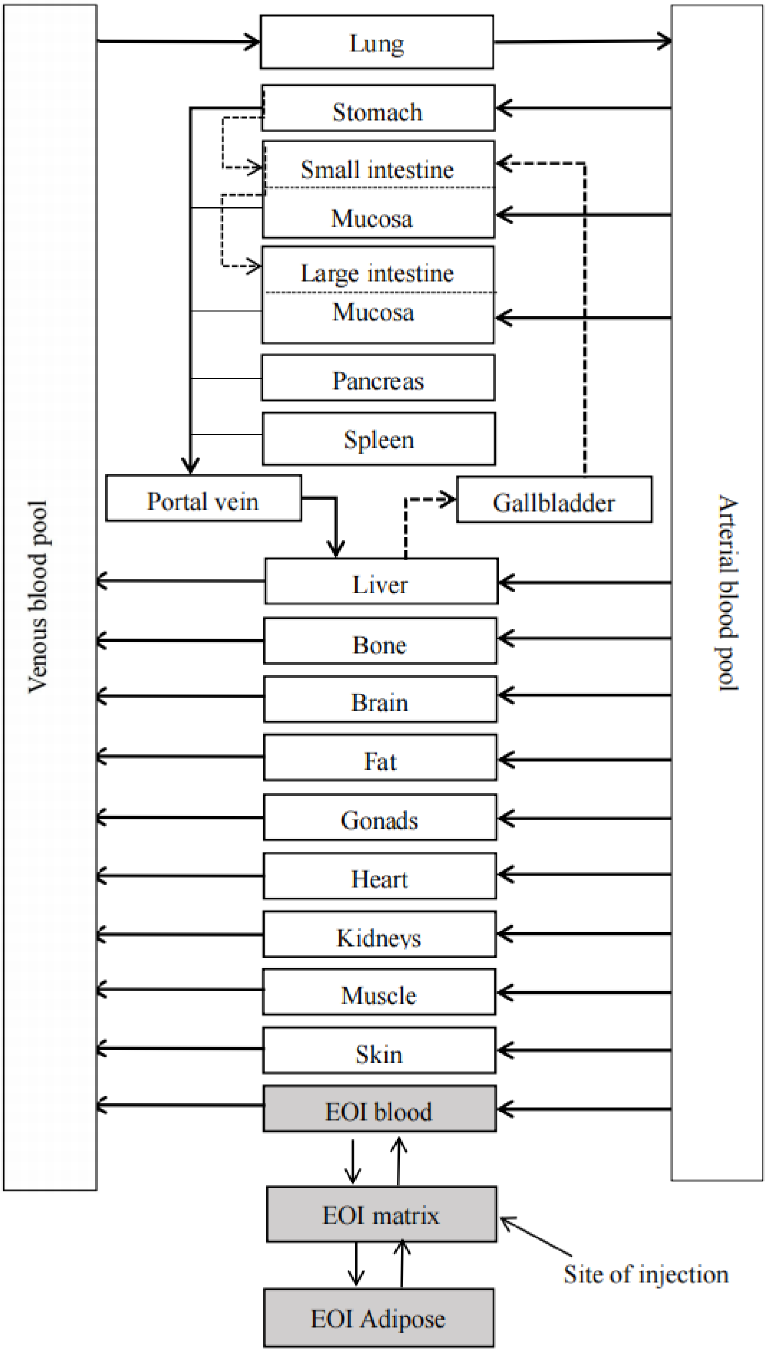

Figure S5 Structure of the PBPK model developed in this paper. The white compartment is the structure of the adult population. Light grey compartments are EOI block dosing structures. Black solid arrows indicate drug transport through the bloodstream. Dashed arrows indicate drug transport through gastrointestinal motility or excretion through the bile. EOI: external oblique intercostal.
