# Supplementary material for: Physiology-Based Pharmacokinetic Modeling of Ropivacaine After External Oblique Intercostal Plane Block in Open Liver Surgery Patients
Source: Pharmaceuticals (Basel). 2026 Feb 24;19(3):348. doi: 10.3390/ph19030348 (PMC13029281; doi:10.3390/ph19030348)
Supplement: Supplementary file 1 [file pharmaceuticals-19-00348-s001.zip › Supplementary File S3 ClinPK checklist.pdf]

| Checklist Item | | |
|---|---|---|
| | **Title/Abstract** | **Reported on Page Number** |
| 1 | The title identifies the drug(s) and patient population(s) studied. | 1 |
| 2 | The abstract minimally includes the name of the drug(s) studied, route of administration, population in whom it was studied, and results of the primary objective and major clinical pharmacokinetic findings. | 1 |
| | **Background** | |
| 3 | Pharmacokinetic data (i.e., absorption, distribution, metabolism, excretion) that are known and relevant to the drugs being studied are described. | 2 |
| 4 | An explanation of the study rationale is provided. | 2 |
| 5 | Specific objectives or hypotheses are provided. | 2 |
| | **Methods** | |
| 6 | Eligibility criteria of study participants are described. | 10 |
| 7 | Co-administration (or lack thereof) of study drug(s) with other potentially interacting drugs or food within this study is described. | 10 |
| 8 | Drug preparation and administration characteristics, including dose, route, formulation, infusion duration (if applicable), and frequency are described. | 10 |
| 9 | Body fluid or tissue sampling (timing, frequency, and storage) for quantitative drug measurement are described. | 10 |
| 10 | Validation of quantitative bioanalytical methods used in the study are referenced or described if applicable. | 10-11 |
| 11 | Pharmacokinetic modeling methods and software used are described, including assumptions made regarding the number of compartments and order of kinetics (zero, first, or mixed order). | 11-12 |
| 12 | For population pharmacokinetic studies, covariates incorporated into pharmacokinetic models are identified and described. | Not applicable |
| 13 | Formulas for calculated variables (such as creatinine clearance, body surface area, AUC, and adjusted body weight) are provided or referenced. | 14 |

| | | |
|---|---|---|
| 14 | The specific body weight used in drug dosing and pharmacokinetic calculations are reported (i.e., ideal body weight vs. actual body weight vs. adjusted body weight). | Not applicable |
| 15 | Statistical methods, including software used, are described. | 14 |
| | Results | |
| 16 | Study withdrawals or subjects lost to follow-up (or lack thereof) are reported. | 3 |
| 17 | Quantification of missing or excluded data is provided if applicable. | 3 |
| 18 | All relevant variables that may explain inter- and intra-patient pharmacokinetic variability (including: age, sex, end-organ function, ethnicity, weight or BMI, health status or severity of illness, and pertinent co-morbidities) are provided with appropriate measures of variance. | 3 |
| 19 | Results of pharmacokinetic analyses are reported with appropriate measures of precision (such as range or 95% confidence intervals). | 4 |
| 20 | Studies in patients receiving extracorporeal drug removal (i.e., dialysis) should report the mode of drug removal, type of filters used, duration of therapy, and relevant flow rates. | Not applicable |
| 21 | In studies of drug bioavailability comparing two formulations of the same drug, F (bioavailability), AUC, $C_{max}$ (maximal concentration), and $t_{max}$ (time to maximal concentration) should be reported. | Not applicable |
| | **Discussion/Conclusion** | |
| 22 | Study limitations describing potential sources of bias and imprecision, where relevant, should be described. | 9 |
| 23 | The relevance of study findings (applicability, external validity) is described. | 8 |
| 24 | Funding sources and conflicts of interest for the authors are disclosed. | 15 |
